# Continuous Monitoring of Muscle Oxygenation in Endurance Athletes During Incremental Cycling: Experimental Validation of a Wearable Continuous-Wave NIRS Sensor Using Frequency-Domain Near-Infrared Spectroscopy

**DOI:** 10.3390/bioengineering12111153

**Published:** 2025-10-24

**Authors:** Evan Peikon, Jennifer L. Corso, Nikola Otic, Olivia Kierul, Maria A. Franceschini, Mitchell Robinson

**Affiliations:** 1 NNOXX, Inc., Seattle, WA 98104, USA; jennifer.corso@nnoxx.com; 2 The Athinoula A. Martinos Center for Biomedical Imaging, Department of Radiology, Massachusetts General Hospital, Harvard Medical School, Charlestown, MA 02129, USA; notic@mgh.harvard.edu (N.O.); okierul@mgb.org (O.K.); mfranceschini@mgh.harvard.edu (M.A.F.); mitchell.robinson@mgh.harvard.edu (M.R.); 3 Department of Biomedical Engineering, Boston University, Boston, MA 02215, USA

**Keywords:** SmO_2_, muscle oxygen saturation, muscle oxygenation, CW-NIRS, continuous-wave NIRS, near-infrared spectroscopy, wearable sensor, FDNIRS, bio-optics, fitness tracker

## Abstract

Individuals often lack field-based tools to monitor exercise effectiveness. New sensing methods may allow for an improved measurement of the individualized response to exercise by monitoring oxygen kinetics directly in muscle tissue. This study aimed to validate a non-invasive wearable sensor capable of measuring muscle oxygen saturation (SmO_2_) using continuous-wave near-infrared spectroscopy (CW-NIRS) against a laboratory-validated frequency-domain NIRS (FDNIRS) device. Ten physically fit adults performed an incremental cycling test until voluntary exhaustion. Devices were placed on contralateral rectus femoris muscles. SmO_2_ was simultaneously measured continuously for the duration of the protocol. Time series alignment was performed using linear interpolation to enable direct comparison between devices at matched time points. Z-score normalization accounted for inter-individual differences in a group-level analysis. Individual subject validation showed strong correlations between the two devices (r = 0.792, range: 0.69–0.88, *p* < 0.001) with an RMSD < 5% for most subjects, a mean bias of 0.005 and low proportional bias (−0.199) between all paired measurements. Group-level analysis demonstrated a correlation of r = 0.788. Bland–Altman analysis revealed that 95% of all measurements fell between −8.1% and 7.6% SmO_2_. The CW-NIRS device delivered reliable performance compared to the FDNIRS device, offering potential applications for real-time physiological monitoring during exercise and performance assessment.

## 1. Introduction

Regular physical activity is essential for improving health, fitness, and physical performance. However, individuals often lack accessible tools to monitor the effectiveness and efficiency of their exercise routines. Current wearable fitness devices primarily track metrics such as heart rate, steps, and estimated caloric expenditure, but these parameters provide limited insight into the actual physiological responses of working muscle during exercise and the related hematological and tissue responses to changing oxygen needs. Additionally, training intensity and adaptation have traditionally been monitored using systemic physiological markers such as heart rate, blood lactate concentration, or oxygen consumption (VO_2_). While these parameters provide valuable insights into overall physiological stress, they offer limited information about the specific metabolic changes occurring within the working muscles themselves.

In recent years, there has been growing interest in muscle oxygen saturation (SmO_2_) monitoring as a direct, localized, and continuous measurement of muscle metabolism and oxygen kinetics during exercise. SmO_2_ represents the balance between oxygen supply and demand within muscle tissue and serves as a valuable indicator of local muscle metabolic activity during physical exertion. Resting muscle oxygen saturation typically ranges from 60–80% in healthy individuals but can vary significantly based on individual factors including training status and disease [1]. During exercise, such as interval training, SmO_2_ values generally decrease as oxygen demand exceeds supply, with the magnitude and pattern of these changes varying considerably between individuals based on their physiological state, fitness level, tissue morphology, and type of exercise being conducted (i.e., steady state aerobic exercise versus resistance training).

Near-infrared spectroscopy (NIRS) has emerged as the primary technology for non-invasive assessment of muscle oxygenation [2,3,4]. NIRS technology utilizes light in the 650–900 nm wavelength range to penetrate tissue and measure the diffused light, allowing estimation of tissue absorption and scattering properties [5,6]. By analyzing the absorption spectrum, researchers can calculate the concentrations of oxygenated and deoxygenated hemoglobin and myoglobin, which are used to calculate SmO_2_ as the ratio of oxygenated hemoglobin and myoglobin to the combined total hemoglobin and myoglobin concentration. 

Multiple studies have investigated the relationship between NIRS-derived parameters, including SmO_2_, and established training metrics. Research has demonstrated correlations between lactate and VO_2_-derived thresholds, and SmO_2_ thresholds identified using NIRS technology [7,8]. During non-steady state activity, changes in SmO_2_ reflect alterations in the balance between oxygen delivery to working muscles and oxygen consumption by mitochondria in working muscles. Other investigations have focused on muscle adaptation throughout training cycles by examining changes in oxygenated hemoglobin, deoxygenated hemoglobin, or muscle oxygenation kinetics during specific exercise protocols [9].

These findings collectively support the integration of NIRS technology into athletic training programs for more targeted and personalized exercise prescription, with a higher personal specificity than typical wearable exercise metrics can provide [10]. It is widely understood that LED-based PPG-derived parameters have technological limitations [11], and SmO_2_ may prove to be a better performance indicator in some use cases [12]. By identifying individual-specific oxygenation thresholds, coaches and athletes can establish precise and more personalized training zones that correspond to distinct metabolic states, enabling more accurate periodization and load management [13]. Furthermore, real-time muscle oxygenation monitoring allows for immediate adjustment of exercise intensity based on localized metabolic demand rather than relying solely on systemic markers, potentially improving training efficiency and reducing the risk of overtraining [14].

Despite these promising applications, traditional muscle oxygenation has been measured using laboratory-based, fiber-coupled NIRS systems, which are expensive, complex to operate, and not suitable for everyday use by the general population or athletes in field settings. The translation of this technology into wearable, wireless formats represents an important advancement that could significantly enhance accessibility and practical application for athletes and coaches, providing real-time feedback during training and competition [15].

To address this technological gap, NNOXX Inc. (Seattle, WA, USA) developed a low cost, wireless, wearable sensor capable of measuring muscle oxygenation and movement acceleration in real-time, with expanded, future capabilities to monitor other hematological parameters [16,17]. It applies a novel deep-optics approach to continuous-wave NIRS (CW-NIRS), utilizing a 3D spectrographic NIRS, improving the ability to discriminately acquire hematological variables in pre-selected tissues of interest. Generally, the design accounts for scattering and absorption coefficients [18], emits light from LEDs between 600–1000 nm and incorporates four separated photodetectors. Photons are segregated by tissue source: those reflected from the skin, subcutaneous adipose layer, muscle tissue, and blood contained in muscle tissue. From a technological perspective, the NNOXX device represents a significant technological advancement in making muscle oxygenation monitoring accessible outside laboratory settings.

FDNIRS systems are one of the most reliable non-invasive devices used to validate tissue oximeters, including muscle oximeters, due to their superior accuracy and ability to separate absorption and scattering effects on tissue measurements [19]. Multiple wearable muscle oximeters, including the Humon Hex, have established their validity through comparison against FDNIRS reference systems like the MetaOx, making this approach an accepted standard for device validation in the field [20]. Unlike traditional FDNIRS systems that require fiber-optic coupling, the NNOXX device employs an optical design optimized for low-power operation while maintaining high signal contrast, eliminating the need for costly modulated light sources and phase-sensitive detection using used in FDNIRS. It instead relies on sophisticated algorithms and optimized hardware to extract physiologically relevant information from simpler optical measurements. The device’s engineering specifically addresses the primary limitations of existing muscle oximeters through integrated ambient light shielding, a convex case back optimized for consistent skin coupling, and on-board signal processing.

A portable CWNIRS device that delivers individualized responses to exercise specifically in local working tissue may be invaluable to applications in cardiovascular rehabilitation or similar because it offers a personalized understanding of exercise efficacy and the efficiency of a workout or series of workouts (training) [10]. By measuring exercise-induced hyperemia and proactive hematological biometrics, exercise prescriptions may be better tailored to a patient’s physiological abilities, improving overall compliance and case management.

This pilot study aimed to validate SmO_2_ measurements obtained from the NNOXX One device against a validated laboratory-standard frequency-domain near-infrared spectroscopy (FDNIRS) device. The specific goal was to determine whether the non-invasive NNOXX technology could recover SmO_2_ measurements within 5% of those detected by the FDNIRS benchtop device during an incremental cycling test.

## 2. Materials and Methods

### 2.1. Study Design and Participants

This study was conducted at the Optics Laboratory at the Martinos Center, Massachusetts General Hospital (MGH), Building 149, 13th St., Charlestown, MA. Ten healthy adults between 18 and 50 years of age who self-identified as endurance athletes were recruited to participate in the study. The study was advertised on Rally, and flyers were posted in hospitals, gyms and campuses in the local Boston area. All participants completed a pre-screening eligibility form via RedCap and provided informed consent before participation. Subjects were required to have participated in endurance training a minimum of 3 times per week. Exclusion criteria included cardiovascular contraindications to exercise, movement disorders, neurological disorders and medications that may impact outcomes of the study, history of hematological disorders, respiratory disease, diabetes and other serious chronic conditions, smoking and substance abuse, pregnancy, BMI > 30 kg/mg^2^, and poor muscle tone in the legs.

### 2.2. Experimental Protocol

Each participant attended a single one-hour session at the laboratory. Upon arrival, study personnel provided detailed explanations of the procedures and conducted training for the participant. Baseline vital signs were assessed, including body temperature, blood pressure, systemic arterial oxygen saturation, and heart rate. These baseline measurements were collected for safety monitoring and participant characterization but were not incorporated into the device validation analysis.

**Figure 1:**
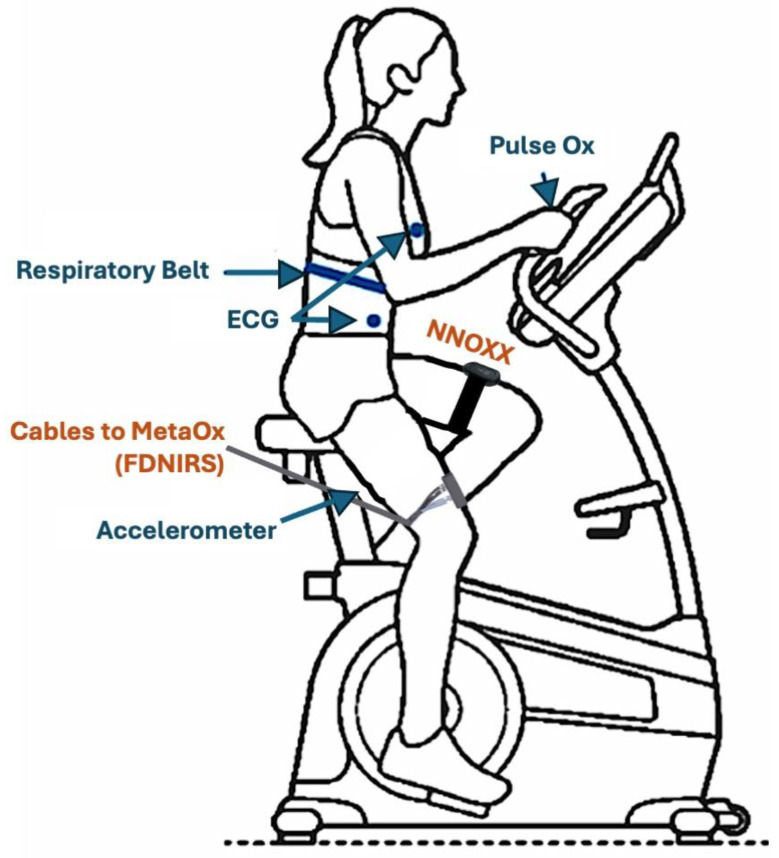
Subject device placement. The FDNIRS device and NNOXX One device were placed on the subject’s rectus femoris, mirroring the position of the other. The NNOXX device was secured to the subject’s leg using a Velcro strap. The FDNIRS device was secured the contralateral leg using adhesive bandages, with the fiber cables lying flat against the leg and out of the way. Additional devices, including an ECG, respiratory belt, fingertip pulse oximeter, and external accelerometer were also placed on the subject as indicated in the image.

The NNOXX One device was attached to the distal head of the rectus femoris muscle, mediolaterally centered with the bottom edge of the device approximately 3.5 inches above the proximal border of the patella (depending on the height of the subject), on either the left or right leg. Devices were counterbalanced across participants, with five having them attached to the left leg and five to the right. The device was oriented vertically with the on/off button proximal to the hip (photodiodes on the case back closest to the knee). The FDNIRS optical probe was symmetrically attached to the rectus femoris muscle on the opposite leg in the anatomical location, horizontally. Fibers and cables were routed to the back of the subject up to the ceiling to minimize interference with movement. See Figure 1 for device placement, below.

The incremental exercise protocol consisted of four distinct stages:

Stage 1: Initial warm-up. Subjects cycled at 100 W for 4 min.

Stage 2: Incremental phase. Power output increased every 4 min (30 W increments for males, 20 W increments for females).

Stage 3: Termination. Criteria for termination was either voluntary exhaustion or reaching a maximum of 9 increments (370 W for males, 280 W for females). 

Stage 4: Cool-down. Subjects cycled at 100 W for 4 min.

Both devices continuously recorded data throughout the exercise test. Data from the NNOXX One was acquired using a mobile phone with NNOXX’s mobile app and exported as CSV files. Data from all other devices was acquired using MGH computers. All devices were manually synchronized at the start of each exercise protocol using visual timing cues.

### 2.3. Devices and Instrumentation

Please refer to Figure 1 for placement of the devices. Two NIRS devices were used to measure changes in muscle hemoglobin saturation:

1. NNOXX One: A continuous-wave, wearable wireless NIRS device provided by NNOXX, Inc. This device utilized LEDs in the red-near-infrared spectral region and four photodiodes as detectors, directly mounted in a battery-operated sensor. The LEDs were turned on and off in rapid sequence, ensuring only one illuminated the tissue at any given time. Data acquisition occurred at 50 Hz sampling frequency.
2. FDNIRS: The MetaOx (ISS Inc., Champaign, IL, USA) device, a validated, bench-top combined frequency-domain NIRS and diffuse-correlation spectroscopy system, was used as the reference device [10]. For this study, only the FDNIRS component was utilized. Eight laser diodes with wavelengths ranging from 670 nm to 840 nm delivered light to the subject via optical fiber bundles. The laser diodes operated sequentially with on-times of 0.01–0.02 s and power less than 5 mW. Light was detected by four detectors through fiber optic cables arranged in a line on the sensor at different distances from the source fiber. The optical fibers are flexible and plug into 90-degree angle prisms so the fibers lie flat against the body. The prisms were arranged on a rectangular rubber sensor of a size no larger than 60 cm^2^ and the sensor was strapped to the subject’s contralateral leg in a mirrored position to the NNOXX device using bandages. The device was controlled by a laptop via a USB cable, and data was acquired at 100 Hz, while the software displayed the acquired data in real time at either 10 or 2 Hz.

Additional physiological parameters were monitored: heart rate via electrocardiogram (ECG); arterial oxygen saturation (SpO_2_) via pulse oximetry; respiratory rate, using a respiratory belt; motion via an external accelerometer placed over the FDNIRS optical sensor.

### 2.4. Data Analysis

Raw (unprocessed) data from the NNOXX One (50 Hz), along with raw and pre-processed data from the FDNIRS device (100 Hz) and systemic physiology data, were processed for analysis. To enable direct comparison between devices with different sampling frequencies, time series data were temporally aligned by first identifying exercise protocol start and end points using accelerometry data. Next, the time axis was normalized from 0.0 to 1.0 for each complete exercise session, then linear interpolation was used to resample the MetaOx data to match the normalized time points of the NNOXX One device, preserving the original number of data points from the NNOXX measurements for paired comparisons.

Statistical analyses included two distinct analytical approaches. For individual-subject validation, we calculated correlation coefficients (Pearson’s r) and root mean square differences (RMSD), defined as the square of the average magnitude of difference between the experimental and reference device using absolute SmO_2_ values to assess device agreement on a per-participant basis.

For group-level analysis, we applied Z-score normalization to each subject’s data independently, transforming individual measurements to have a mean of 0 and standard deviation of 1, before pooling data across all subjects. This normalization approach isolates device agreement from inter-individual physiological variability. While both devices measure absolute SmO_2_ values, individual subjects naturally exhibit different resting (e.g., baseline) SmO_2_ values and response magnitudes during exercise, in majority due to differences in tissue properties and fitness levels. The devices can then be assessed for tracking relative changes within each individual consistently, rather than being confounded by absolute value differences between subjects, thus focusing the validation on device concordance in detecting physiological patterns. While individual subject correlations provide valuable insights into device agreement on a per-participant basis, comprehensive validation required assessment of overall performance across the entire study population.

Group-level normalization enabled meaningful comparison between individuals with different physiological fitness levels and tissue characteristics. For example, a highly trained endurance athlete may demonstrate different baseline SmO_2_ values, greater absolute ranges of change, and distinct temporal patterns compared to a recreationally fit cyclist. These differences reflect genuine physiological adaptations including enhanced capillary density, improved mitochondrial function, and optimized oxygen extraction efficiency in trained individuals. Additionally, tissue-related factors such as subcutaneous adipose thickness, skin pigmentation, and muscle fiber composition contribute to inter-individual variability in absolute SmO_2_ measurements, even when using identical measurement techniques.

Following normalization, we performed a group-level (pooled) correlation analysis. To comprehensively assess the agreement between the NNOXX One and FDNIRS devices, we conducted a Bland–Altman analysis, which is widely recognized as the gold standard statistical method for evaluating the concordance between two measurement techniques. The analysis focuses on the differences between paired measurements plotted against their mean values, providing insights not always apparent from correlation coefficients alone. We also calculated constant bias, proportional bias, and mean bias metrics.

The study was approved by the Mass General Brigham Institutional Review Board (MGB IRB, protocol number 2024P001201). All study data are de-identified.

Generative artificial intelligence (AI) was not used in this paper to generate text, data, or graphics or to assist in study design, data collection, analysis, or interpretation.

## 3. Results

Please refer to Table 1 for subject demographics. The original cohort included 10 subjects completing the protocol. Data from 2 subjects were excluded due to device firmware or file corruption.

### 3.1. Individual Subject Correlations

Strong correlations were observed between the NNOXX One and FDNIRS devices across all eight participants (refer to Table 2). The correlation coefficients (r) ranged from 0.69 to 0.88, with a mean correlation of 0.79. The root mean square difference (RMSD) between devices was 3.5% SmO_2_ (1.2–7.5%), with most subjects showing differences below 5%.

**Figure 2:**
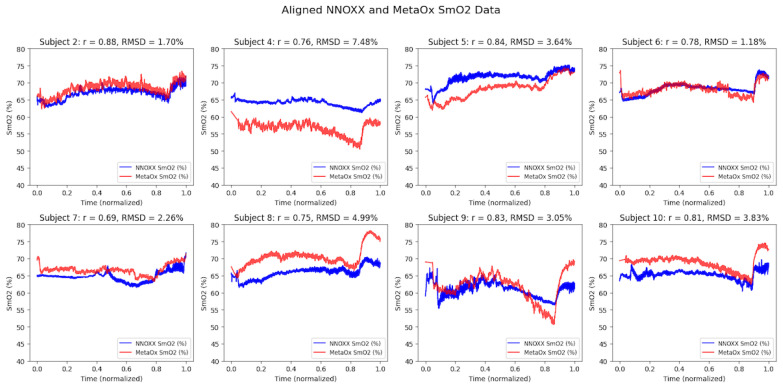
Pairwise comparisons between SmO_2_ readings for the NNOXX CW-NIRS and MetaOx FD-NIRS devices for all eight subjects included in the analysis. Results indicate strong consistency between devices within subjects. Time series data were temporally aligned by normalizing the time axis from 0.0 to 1.0 for each complete exercise session, then resampling the NNOXX One and MetaOx datasets to the matched time points for paired comparisons, enabling direct comparison between devices with different sampling frequencies. Inter-device correlations within subjects range from r = 0.69 to r = 0.88 as explained by Table 2.

Time series plots of aligned data from both devices showed similar patterns of SmO_2_ changes during the incremental exercise protocol (Figure 2). While some inter-subject variability was observed in baseline values and response magnitudes, the temporal patterns and directional changes were highly consistent between devices within each subject.

### 3.2. Group-Level Analysis

Z-score normalization transformed each subject’s measurements independently to have a mean of 0 and a standard deviation of 1, allowing for meaningful comparison and aggregation of data across different individuals. This normalization technique was essential because subjects naturally presented different baseline SmO_2_ levels and ranges of values throughout the exercise protocol. Without normalization, data from subjects with higher absolute values or greater variability would disproportionately influence the group results, potentially skewing the overall assessment of device agreement. The z-score approach preserved the relative patterns of change within each individual while enabling statistical comparison across the diverse physiological characteristics represented in our study population.

Data was then pooled to calculate a group correlation coefficient. The analysis revealed a strong positive correlation of r = 0.788 between the NNOXX One and FDNIRS measurements. The result indicates a substantial agreement between the two devices at the population level, suggesting that the NNOXX One effectively captures the same physiological signal as the reference FDNIRS device across different individuals.

To further characterize the relationship between the two measurement systems, we calculated several bias metrics:

Constant bias: 0.021 (z-score units). This small positive value indicates a minimal fixed offset between the two devices across all measurements, suggesting that one device does not consistently read higher or lower than the other by a substantial margin.

Proportional bias: −0.199. This negative value indicates that the difference between devices slightly varies depending on the magnitude of the measurement. Specifically, the NNOXX One tends to read slightly higher than the FDNIRS at lower SmO_2_ values and slightly lower at higher SmO_2_ values. However, the magnitude of this proportional bias is relatively small and unlikely to significantly impact practical applications.

Mean bias: 0.005 (z-score units). This value represents the average difference between all paired measurements. The result is extremely close to zero, further confirming reliable, overall agreement between the devices.

**Figure 3:**
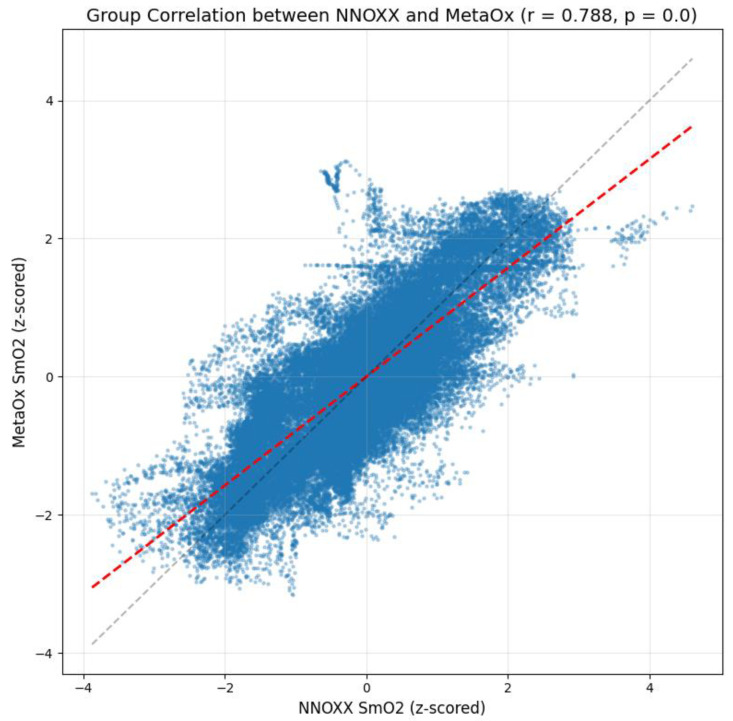
Group correlation between NNOXX CW-NIRS and MetaOx FDNIRS devices. The gray line indicates the line of perfect agreement (y = x), and the red line indicates the regression line. The z-scored measurements of each device were respectively pooled and compared, with a result of r = 0.788. This result indicates a strong correlation between the two devices. A low mean bias of 0.005 between all paired measurements further supports an overall agreement between devices, and the deviation between the regression line and line of perfect agreement indicates a proportional bias of −0.199.

Figure 3 provides a scatter plot of the z-scored NNOXX One measurements against the z-scored FDNIRS measurements. The plot displays a clear positive linear relationship between the two devices with data points clustered along the regression line (red dashed line). The gray dashed line represents the line of perfect agreement (y = x). The deviation between these two lines visualizes the small proportional bias detected in our analysis.

The density of points in the central region of the plot indicates that most measurements fall within typical physiological ranges during the exercise protocol. The spread of data points around the regression line illustrates measurement variability that may be attributed to factors such as small differences in sensor placement, minor temporal misalignments between devices, or inherent limitations of the optical measurement techniques.

### 3.3. Bland–Altman Analysis

**Figure 4:**
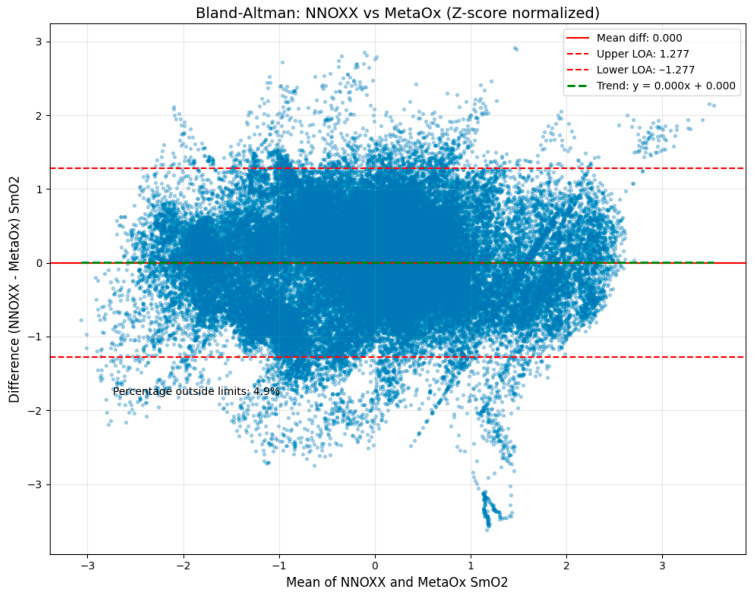
Z-score normalized Bland–Altman plot comparing the NNOXX One and MetaOx devices for all combined data points between the two devices. Results indicate 93.4% of the data points fell within the normalized limits of agreement, between −1.28 and 1.28 units of SmO_2_. This equates to −8.1% to 7.6% SmO_2_ when converted to the original scale, which are narrow limits of agreement when compared to the performance of similar wearable devices.

The Bland–Altman analysis (Figure 4) revealed strong agreement between the two devices with the following key metrics:

Mean difference (bias): 0.000 (z-scored data). This value represents the average difference between all NNOXX One and FDNIRS measurements. The zero bias indicates that, on average, there is no systematic difference between the two devices, suggesting neither device consistently overestimates or underestimates SmO_2_ relative to the other.

Standard deviation of differences: 0.652. This metric quantifies the spread of the differences between paired measurements. The relatively low value indicates good precision and consistency between the two devices across the measurement range.

Upper limit of agreement: 1.277. This value, calculated as the mean difference plus 1.96 standard deviations, represents the upper boundary within which 95% of the differences between measurements are expected to fall.

Lower limit of agreement: −1.277. Similarly, this value (mean difference minus 1.96 standard deviations) represents the lower boundary of the 95% confidence interval for measurement differences.

Figure 4 presents the Bland–Altman plot illustrating differences between NNOXX One and FDNIRS measurements (y-axis) plotted against the mean of both measurements (x-axis). The solid red horizontal line represents the mean difference (bias), while the upper and lower dashed red lines indicate the limits of agreement. The green dashed horizontal line represents the trend of the differences across the measurement range.

This analysis indicates that 95% of the differences between measurements were expected to fall between −1.28 and 1.28 in normalized units, which corresponds to approximately −8.1% to 7.6% in SmO_2_ units when converted back to the original scale. This range is notably narrow for physiological measurements, particularly for wearable technology, supporting strong agreement between the devices.

Importantly, only 6.6% of all data points fell outside these limits of agreement, which is close to the expected 5% for normally distributed differences. This finding further validates the statistical robustness of our analysis and confirms the consistency of agreement across the measurement range.

A small proportional bias was observed, with a trend line slope of −0.550, indicating that the NNOXX device tended to read slightly higher than the FDNIRS at lower oxygen saturation values and slightly lower at higher values. This pattern may be attributed to differences in the underlying technology (continuous-wave versus frequency-domain NIRS) or variations in the algorithms used to calculate SmO_2_. However, this bias was minimal and did not significantly impact the overall agreement between devices.

## 4. Discussion

This study aimed to validate the NNOXX One wearable sensor against a benchtop reference standard FDNIRS device for measuring muscle oxygen saturation during incremental cycling exercise. The results demonstrated strong correlations and excellent agreement between the two devices, supporting the validity of the NNOXX One for non-invasive, real-time monitoring of tissue oxygen saturation in skeletal muscle.

Overall, the group-level analysis demonstrates that, when appropriately normalized to account for individual differences, NNOXX One SmO_2_ measurements strongly correlate with those from the reference standard FDNIRS device. The individual subject correlations indicate that NNOXX One captures the same physiological signals as the reference device, despite differences in technology and limb placement. The RMSD values below 5% for most subjects meet our predefined goal of measuring SmO_2_ changes within 5% of the reference device. This level of accuracy is remarkable considering the technical challenges associated with wearable optical sensing, including motion artifacts, skin pigmentation variations, and subcutaneous fat layer differences [21,22,23,24,25]. The results support the validity of the device for capturing the same physiological signals as the reference during incremental exercise.

The Bland–Altman analysis confirmed the strong agreement between devices with minimal bias. The dense cloud of data points around the zero-difference line throughout the physiologically relevant measurement range demonstrates consistent agreement across typical SmO_2_ values encountered during incremental exercise. The wider spread of points at the measurement range extremes is a common observation in method comparison studies and likely reflects the greater measurement challenges at physiological boundaries. The narrow limits of agreement suggest that the NNOXX One provides clinically meaningful measurements that are comparable to laboratory-grade equipment. The small proportional bias observed could potentially be addressed through calibration adjustments in future iterations of the device. Overall, the Bland–Altman analysis confirms that the NNOXX One provides reliable SmO_2_ measurements across the physiological range typically encountered during exercise, supporting its validity for field applications in sports performance monitoring.

The study design, which included simultaneous bilateral measurements during a standardized incremental exercise protocol, allowed for robust comparison while minimizing confounding factors. By testing the devices during actual exercise conditions rather than in resting states alone, we were able to validate performance across a physiologically relevant range of muscle oxygen saturation levels.

Several limitations should be acknowledged. First, the sample size of ten participants, with only eight subjects having usable data due to file corruption while downloading raw data from the NNOXX prototype device, while sufficient for an initial validation study, may limit generalizability.

Second, the devices show slightly different response magnitudes during certain phases of the exercise protocol, with instances where one device exhibits a larger amplitude change than the other despite tracking similar directional trends. This may reflect inherent differences between CW- and FDNIRS technologies, which employ different optical approaches. Additionally, the contralateral placement necessitated by the fiber-coupled reference system introduces physiological variability between measurement sites that may contribute to these observed differences [26]. Muscle oxygenation signals are inherently variable due to multiple factors including device placement precision, local tissue heterogeneity, and subtle differences in muscle fiber recruitment between limbs. Even during symmetrical exercise like cycling, small biomechanical differences between legs, varying degrees of muscle fatigue, and individual anatomical asymmetries can contribute to measurement discrepancies between devices placed on opposite limbs.

While the devices demonstrate strong overall correlations and clinically acceptable agreement levels, we recognize that the magnitude and timing differences observed could impact real-time monitoring applications where precise kinetic matching is critical. However, for many practical applications in exercise monitoring, the ability to track relative changes and directional patterns may be sufficient for training guidance, as evidenced by the robust statistical agreement demonstrated across our validation metrics.

Third, all participants were healthy, physically fit individuals. Results may differ in clinical populations or those with altered muscle physiology, as well as variations in adiposity [27,28,29]. Subcutaneous adipose tissue thickness was not measured in this study, which represents a limitation as tissue thickness can influence NIRS signal quality and penetration depth [30]. This is particularly relevant given that 70% of participants (7 out of 10) were female athletes who may have different adipose distributions compared to males. The subject demographics lend a unique perspective to the validation outcomes, and comparatively, to similar tissue oximeter studies that are typically male dominant [14].

Fourth, measurements were collected only during cycling exercise, which also limits translation of the data to other types of exercise, such as resistance training. Validation during other exercise modalities would strengthen confidence in the device’s versatility across different movement patterns and muscle recruitment strategies.

Finally, an important methodological consideration in this study was the choice of the rectus femoris muscle for sensor placement rather than the vastus lateralis, which is recognized as the primary single-joint muscle and prime mover during cycling exercise [31]. This muscle selection was chosen due to logistical constraints related to the fiber-coupled reference FDNIRS system, which required specific positioning and cable routing considerations to minimize interference during the pedaling motion. While this placement choice may have resulted in less pronounced SmO_2_ changes compared to measurements from the vastus lateralis, both muscles are active during cycling, and the consistent bilateral placement approach ensured comparable measurement conditions between devices.

Future research should expand validation to diverse populations, including clinical groups and individuals with varying fitness levels. Additional exercise modalities, including asymmetrical movements and resistance training, should be tested to further establish validity across exercise contexts. Longitudinal studies exploring the relationship between muscle oxygenation patterns and training adaptations would help establish the practical utility of this technology for optimizing exercise prescriptions.

## 5. Conclusions

The NNOXX One wearable sensor demonstrates strong validity for non-invasive measurement of muscle oxygen saturation during exercise when compared to a benchtop reference standard FDNIRS device. With strong correlation coefficients and measurement differences between devices generally below 5%, the device fulfills the predefined criteria for acceptable agreement. The minimal bias and narrow limits of agreement observed in the Bland–Altman analysis further support its reliability, as does the small proportional bias observed, which is unlikely to have meaningful practical implications for training applications.

This validation provides a foundation for the use of wearable muscle oxygenation monitoring in real-world settings, potentially transforming how individuals and athletes monitor and optimize their training, and offers a reliable springboard for further sensor development. By providing accessible insights into muscle metabolism during exercise, this technology may enable more targeted and efficient training strategies, ultimately enhancing performance outcomes and exercise-related health benefits.

Furthermore, the NNOXX One represents a significant advancement in wearable fitness technology, moving beyond basic metrics to provide deeper physiological insights that were previously only available in laboratory settings. This bridge between benchtop performance and real-world applicability opens new possibilities for research, athletic training, and personalized exercise prescription in clinical settings.

## 6. Patents

NNOXX Inc. holds IP related to the device design and clinical applications of the optics and platform [32,33,34,35,36].

## Figures and Tables

**Table 1:** Demographic and anthropometric subject characteristics.

	Total n	Male	Female
Subjects (n)	10	3	7
Right-footed	9	3	6
Age (years)	33 ± 8	32 ± 7	33 ± 9
Weight (kg)	68 ± 12	80 ± 16	63 ± 7
Height (cm)	168 ± 10	180 ± 10	165 ± 8
BMI (kg/m^2^)	24 ± 3	25 ± 4	24 ± 2

**Table 2:** Mean SmO_2_ measurements per subject per device, and individual subject correlations between the NNOXX CW-NIRS device and MetaOx FDNIRS reference device. The correlation coefficient is the average of the entire exercise protocol (e.g., Incremental cycling and cool-down). The correlation values ranged between 0.69 to 0.88 with a mean of 0.79, with an RMSD ranging from 0.012 to 0.075 (1.2% to 7.5%), which indicates a strong agreement between the devices with a low average difference.

Subject	MetaOx SmO_2_ (Mean %)	StDev (%)	NNOXX One SmO_2_ (Mean %)	StDev (%)	Correlation (r)	RMSD
2	68.4	1.9	66.9	1.8	0.88	0.017 (1.7%)
4	56.7	2.0	64.0	1.0	0.76	0.075 (7.5%)
5	68.1	3.0	71.4	2.0	0.84	0.036 (3.6%)
6	68.1	1.8	68.2	1.8	0.78	0.012 (1.2%)
7	66.7	1.4	64.7	1.5	0.69	0.023 (2.3%)
8	70.5	2.8	65.9	1.7	0.75	0.050 (5.0%)
9	62.0	4.3	60.9	2.1	0.83	0.031 (3.1%)
10	68.8	2.3	65.3	1.3	0.81	0.038 (3.8%)

## Data Availability

Data presented in this study are available on request from the corresponding author for IP reasons.

## Abbreviations

The following abbreviations are used in this manuscript:

CW-NIRS	Continuous-wave near-infrared spectroscopy
ECG	Electrocardiograph
FDNIRS	Frequency-domain near-infrared spectroscopy
HR	Heart rate
LED	Light-emitting diode
MEMS	Microelectromechanical System
NIRS	Near-infrared spectroscopy
SmO_2_	Muscle oxygen saturation
SNO	S-nitrosothiol
SpO_2_	Peripheral blood oxygen saturation
VO_2_	Oxygen consumption (volume of oxygen consumed)
VO_2_max	Maximal oxygen consumption
